# Perspectives Regarding Medications for Opioid Use Disorder Among Individuals with Mental Illness

**DOI:** 10.1007/s10597-022-01012-x

**Published:** 2022-07-29

**Authors:** Derjung M. Tarn, Kevin J. Shih, Allison J. Ober, Sarah B. Hunter, Katherine E. Watkins, Jeremy Martinez, Alanna Montero, Michael McCreary, Isabel Leamon, John Sheehe, Elizabeth Bromley

**Affiliations:** 1 Department of Family Medicine, University of California, Los Angeles, Los Angeles, CA USA; 2 RAND Corporation, Santa Monica, CA USA; 3 LA County Department of Mental Health, Los Angeles, USA; 4 Department of Psychiatry and Biobehavioral Sciences, University of California, Los Angeles, Los Angeles, CA USA; 5 Department of Family Medicine, David Geffen School of Medicine at UCLA, 10880 Wilshire Blvd., Suite 1800, Los Angeles, CA 90024 USA

**Keywords:** Mental disorders, Opioid use disorders, Opioid-related disorders, Narcotic antagonists, Buprenorphine-naloxone drug combination, Qualitative research

## Abstract

Most people with co-occurring opioid use disorder (OUD) and mental illness do not receive effective medications for treating OUD. To investigate perspectives of adults in a publicly-funded mental health system regarding medications for OUD (MOUD), we conducted semi-structured telephone interviews with 13 adults with OUD (current or previous diagnosis) receiving mental health treatment. Themes that emerged included: perceiving or using MOUDs as a substitute for opioids or a temporary solution to prevent withdrawal symptoms; negative perceptions about methadone/methadone clinics; and viewing MOUD use as “cheating”. Readiness to quit was important for patients to consider MOUDs. All participants were receptive to discussing MOUDs with their mental health providers and welcomed the convenience of receiving care for their mental health and OUD at the same location. In conclusion, clients at publicly-funded mental health clinics support MOUD treatment, signaling a need to expand access and build awareness of MOUDs in these settings.

## Introduction

Mental illness and substance use disorders co-occur in about 9.5 million adults in the United States, yet many of these individuals have an untreated substance use disorder (Harbaugh et al., 2018; Reid & Palamar, 2021; Substance Abuse & Mental Health Services Administration, 2019). Untreated opioid use disorders (OUD) can result in devastating consequences. In Los Angeles County alone opioid overdoses increased by 467% from 2015 to 2020 (California Department of Public Health Substance and Addiction Prevention Branch). People with both mental illness and a substance use disorder (commonly referred to as a co-occurring disorder) are at greater risk for adverse OUD-related outcomes such as homelessness, incarceration, and suicide than those with only a single disorder (Ashrafioun et al., 2020; Manhapra et al., 2021; Winter et al., 2019). These individuals more often present and receive care in mental health systems rather than in addiction medicine clinics (Harris & Edlund, 2005; Simpson et al., 2020; Watkins et al., 2001), but unfortunately almost 80% of adults with co-occurring mental illness and OUD presenting to public mental health clinics have never received substance use treatment (Ober et al., 2021).

FDA-approved medications for OUD (MOUD) such as buprenorphine and naltrexone are effective as therapeutic approaches aimed at lessening and discontinuing problematic opioid use (Baser et al., 2011; Gerra et al., 2006; Schackman et al., 2012), but have not been widely adopted (Hawk et al., 2020; Moore et al., 2019; Morgan et al., 2018; Substance Abuse & Mental Health Services Administration, 2019). Population-level data may be critical for assessing the needs of people with co-occurring disorders and to narrow treatment gaps (U.S. Department of Health & Human Services, 2019). However, at the individual level, little knowledge exists regarding how those with co-occurring disorders perceive MOUDs. Existing studies on perceptions about MOUDs have not focused on these populations (Cioe et al., 2020; Oliva et al., 2011; Randall-Kosich et al., 2020; Silverstein et al., 2019; Yarborough et al., 2016). Patient attitudes and preferences are important determinants of adherence to medications used to treat mental disorders, and consequently of treatment outcomes (Budd et al., 1996; Bussing et al., 2012; Cabeza et al., 2000; Svedberg et al., 2003). Thus, given policy recommendations to increase access to MOUD for OUD (Hedberg et al., 2019; Jones, 2013; Kolodny & Frieden, 2017), there is an urgent need to understand the perspectives of those with OUD who are accessing public mental health care.

**Fig. 1:**
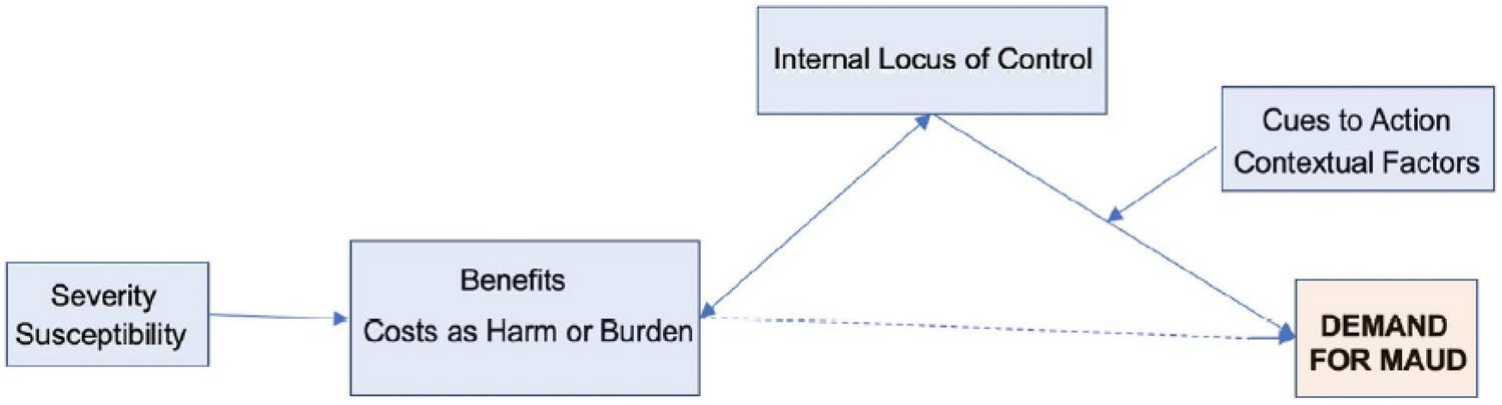
Health Belief Model, adapted for medications for substance use disorders (Bromley et al., 2020)

A previously developed framework adapted from the Health Belief Model (HBM) (Becker, 1974; Strecher & Rosenstock, 1997) may help to inform us how individual perceptions result in demand for (or use of) MOUD (Fig. 1). This framework focused on drivers of clients’ likelihood to use medications for alcohol use disorder (MAUD) (Bromley et al., 2020). It posited that people base decisions about treatment on their perceptions of their disease *severity* and *susceptibility* to adverse outcomes, as well as the *costs* (i.e., harms or burdens) of the treatment. In the framework, the relationship of these constructs and MAUD demand was strongly mediated by an individual’s beliefs regarding their control over their health outcomes (*locus of control*). Locus of control can be internal (based on own actions), external (based on events outside of one’s own control) or related to chance. Those with a strong internal locus of control may consider self-control to be most important for quitting opioids, compared to those whose locus of control is situated externally or related to chance. *Cues to action* (a stimulus or prompt that triggers decision-making, such as advice from others) and *contextual* factors (environmental conditions such as feelings of stigma) may prompt decisions about treatments.

The objective of this study is to examine perspectives of patients in the public mental health system with co-occurring mental health and OUD regarding MOUD treatment. We use the HBM that was adapted to understand patient perspectives of MAUD as a framework to understand how perceptions elicited during semi-structured interviews may drive demand for MOUD (Becker, 1974; Strecher & Rosenstock, 1997).

## Methods

### Study Setting and Participants

We conducted semi-structured interviews with adults receiving care at Los Angeles County Department of Mental Health (LACDMH) outpatient clinics as part of a larger study to develop a toolkit to increase MOUD uptake in public mental health settings (Watkins et al., 2021). Public mental health clinics are unique because they provide healthcare predominantly to low-income and uninsured patients. The clinics we selected for recruitment were located in each of Los Angeles county’s eight service planning areas. Patient populations at the participating sites reflected the ethnic, racial and geographic diversity of populations in Los Angeles County (Ober et al., 2021). These included both large and small clinics, as well as semi-rural and urban sites. All clients who met eligibility criteria were invited to participate. The clinics provided varying levels of substance use disorder counseling but although they have high prevalence of OUD, they have low rates of MOUD prescribing. Study investigators contacted LACDMH clinic program directors to describe the study opportunity and request that staff refer eligible individuals. Supervisors and clinicians at six clinics identified and referred eligible individuals to the investigative team for the interviews. Eligible clients were those with a history of opioid use disorder (heroin and/or prescription opioids) and mental illness who were being treated at one of the participating clinics. They did not need to have experience with MOUD. Individuals provided verbal informed consent prior to the interviews and received a $30 gift card for their participation.

### Data Collection

Participants completed one-time 45–60-min interviews by telephone between October 2020 and March 2021. Interviewers used a semi-structured interview guide to ask clients about their conversations with LACDMH providers regarding their OUD, experiences with MOUDs, knowledge and preferences regarding MOUDs, and attitudes towards receiving OUD treatment in the mental health setting (Table 1).

**Table 1:** Participant characteristics (n = 13)

Characteristic	n (%) or mean (SD)
Age in years, mean (SD; range)	47.8 (12.4; 28–66)
Female, n (%)	5 (38.5)
Race, n (%)	
Hispanic White	6 (46.2)
Black or African American	3 (23.0)
Non-Hispanic White	2 (15.4)
More than one race	2 (15.4)
Psychiatric diagnosis^1^, n (%)	
ADHD	1 (7.7)
Bipolar disorder	5 (38.5)
Major depressive disorder	11 (84.6)
Other anxiety disorder	7 (53.8)
PTSD	3 (23.1)
Schizophrenia	3 (23.1)
Told by doctor that they have OUD, n (%)	7 (53.8)
Prescription opioid misuse over the past 12 months, n (%)	
Never	6 (46.2)
1 time per month or less	1 (7.7)
2–4 times a month	2 (15.4)
2–3 times a week	1 (7.7)
4 or more times a week	3 (23.1)
Heroin use over the past 12 months, n (%)	
Never	7 (53.8)
1 time per month or less	2 (15.4)
2–4 times a month	0 (0.0)
2–3 times a week	0 (0.0)
4 or more times a week	4 (30.8)
Intention to quit opioid medication or heroin, n (%)	
Quit	7 (53.8)
Trying to quit	1 (7.7)
Considering trying to quit	4 (30.8)
Not trying to quit	1 (7.7)
Other substance use over the past 12 months^1^, n (%)	
Marijuana	9 (69.2)
Methamphetamine	7 (53.8)
Amphetamine-type stimulants	5 (38.5)
Powder cocaine or crack	4 (30.8)
Sedatives or sleeping pills	4 (30.8)
Preferred method of MOUD administration^1^, n (%)	
Injection	6 (46.2)
Oral	5 (38.5)
Sublingual	4 (30.8)

^1^Percentages add up to more than 100% because multiple selections were allowed

The interview guide was based on constructs associated with the Health Belief Model (HBM), a model widely used to understand individual health behaviors (Becker, 1974; Strecher & Rosenstock, 1997), and more specifically on the HBM adapted to understand MAUD (Bromley et al., 2020). All interviews were audio-recorded. KJS (a sociologist) conducted all semi-structured interviews, and generated field notes after each interview. DMT (a physician-investigator) co-led three-fourths of the interviews. The two investigators discussed the interview content and reviewed emerging themes after each interview. To better solicit client explanations, the two investigators adjusted the interview guide as needed.

Immediately after completing their interview, clients responded to questions about their demographics, psychiatric diagnoses and experiences with opioids, MOUDs and other substances.

### Qualitative Analysis

All interviews were transcribed verbatim and de-identified. ATLAS.ti 9 was used for analysis. Using notes from discussions after each interview and repeated review of all transcripts, KJS and DMT developed a preliminary set of codes to mark relevant text addressing participant perspectives regarding MOUD. Both then independently applied codes to 4 new transcripts, resolved discrepancies in code use through consensus, and refined and finalized a codebook to capture content relevant to MOUD perceptions. Next, KJS coded all and DMT coded half of the remaining transcripts, comparing their coding to ensure consistent application of the codes and to resolve discrepancies. Once all of the interviews were coded, KJS and DMT used an inductive thematic approach to group codes and synthesize groups of codes in order to elaborate themes pertaining to perceptions regarding MOUD and preferences for receiving MOUD in a mental health clinic (Braun & Clarke, 2006; Braun et al., 2019; Joffe, 2011). They then compared elaborated themes against constructs of the HBM to explore relationships between and among themes and to examine how themes relate to decision-making about MOUD. Major themes were those that more than half of participants mentioned, and minor themes were ones mentioned by fewer than half. Consensus on the themes was reached with the entire investigative team.

This study was approved by RAND’s institutional review board (IRB) and the Los Angeles County Department of Mental Health Human Subjects Research Committee. All authors certify responsibility for this study.

## Results

**Fig. 2:**
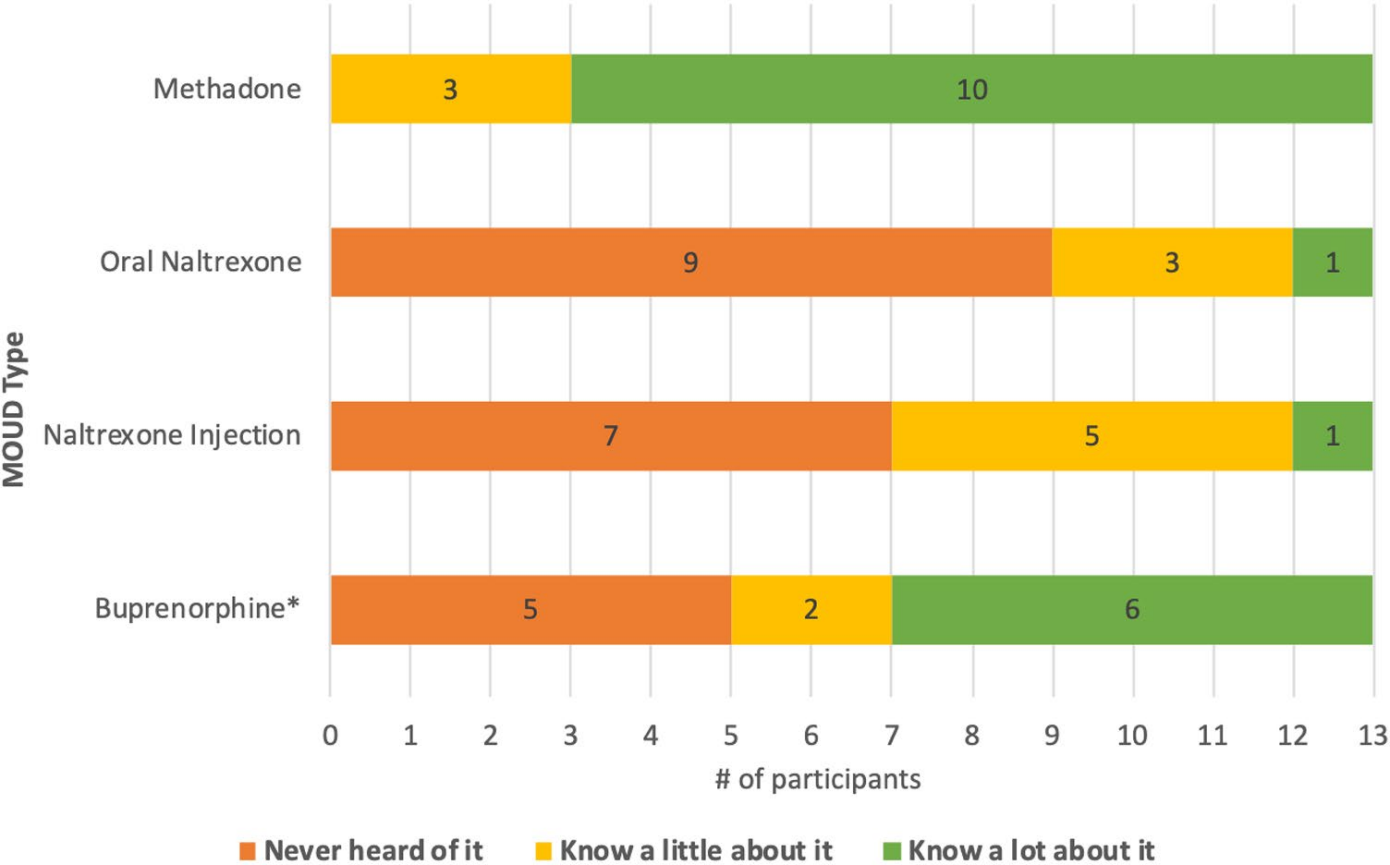
Participant knowledge of different types of MOUDs. *Participants were asked about “buprenorphine, Suboxone or Subutex” (Color figure online)

Of 21 clients who were referred to or contacted the investigative team, 2 were ineligible because they had no history of opioid use, 3 were referred but never contacted the investigators, 3 contacted the investigators but subsequently did not respond to investigator phone calls, and 13 participated in the interviews. Of these, about one-third were female, the mean age was 47.8 (SD = 12.4), almost all reported having major depressive disorder and over half reported having other anxiety disorder (Table 1). The majority had multiple diagnoses. Over three-fourths of the participants had a history of both heroin and prescription opioid use, but 5 (38%) had not used any opioids in the past 12 months. All participants knew a little about methadone, but fewer had knowledge about other MOUDS (buprenorphine, oral naltrexone, naltrexone injection); the majority had never heard of oral or injectable naltrexone (Fig. 2). Below we describe major themes corresponding to HBM constructs. Results are organized by the HBM constructs noted in Fig. 1. Additional examples along with minor themes are illustrated in Table 2.

**Table 2:** Health Belief Model Constructs, associated major and minor themes and illustrative quotes

Health belief model construct	Major and minor themes identified in data	Sample quotes
Severity/susceptibility	Readiness to quit is critical for consideration of MOUDs	“[For someone to be receptive of MOUDs] it has to be somebody that’s really determined to get sober” [Participant 13]
		“One reason [that addicts would not want to take an MOUD] is they just don’t want to stop. They like having a high feeling and taking that next score” [Participant 4]
Benefits	MOUDs are used to substitute for opioids or stave off withdrawals, not for OUD treatment	“I started using Suboxone, I was getting off the streets. All the [MOUDs] you mentioned, they sell them on the streets as dope, because you get high off them. I had the desire to stop the dope, and I started buying the Suboxone, doing it and everything but the problem is, I started getting abuse” [Participant 13]
		“[Using methadone is] not to avoid [opioids], they just do it for the high” [Participant 7]
		“[Addicts around me] would take [methadone] and still go use [opioids] a little bit. It’s like they were matching their personal use to get through the day” [Participant 2]
	MOUDs can assist with withdrawals	“The flip side of that would be these medications are helping opiate users not feel the withdrawal. Therefore, withdrawing becomes not a battle… Once you go through [withdrawals], you don’t want to go through that again” [Participant 3]
	Decreased pill burden of MOUDs is desirable	“[The doctor] said, “I have something that is long-acting. That you won’t have to take so many pills. That you will be not 100% pain-free but you will be in a comfortable place as to where you can function for 12 hours”. That sounds good to me. I don’t have to take all of these pills. At some point in time, they stop working so you just needed more” [Participant 1]
	MOUDs are not effective for achieving sobriety	“After I discovered Suboxone, I was like, “Oh, no. This is such a crutch. No one’s going to get sober like that”. They’ll take it one day and be sober maybe for that day, but they don’t feel the withdrawal, so they go on to using the very next day” [Participant 3]
		“I was buying [methadone] off the street to help me, and it wasn’t doing anything. That’s why I was like, “Oh, that’s not going to help me [quit] either” [Participant 9]
Costs (as harms or burden)	Negative perceptions about methadone and methadone clinics are prevalent	“Methadone is bad for people, I think it’s worse than heroin. Because I feel like people get worse with that…You know, from your bones, you lose your teeth, you lose everything. You know what I mean?” [Participant 12]
		“I feel like sometimes the staff [at the methadone clinic] is not knowledgeable on the way that [an addict] might feel. I would just [see the staff] toy with their addiction, and that really makes you not want to go back there or even try alternative or a plan to stop. It would actually make you want to just use more” [Participant 6]
	Concerns about interactions between psychiatric medications and MOUDs	“Well, it’s better if they tell me if it’s okay to take with the mental medication that I’m taking right now, the milligrams…That’s the main important thing. Don’t take my mental medication away for this medication. No, that’s not going to work” [Participant 7]
	Potential MOUD side effects	“I got a bad liver and I don’t want to take something that’s going to harm me even more if I’m going through treatment” [Participant 9]
	Need for ongoing use of MOUDs is undesirable	“When I was coming up, Methadone was only issued for two or three weeks just to wean you. Now, these people get it all the time. I totally don’t like that” [Participant 11]
Locus of control	MOUD use feels like “cheating”	“Well, I think that [taking MOUDs] would show a sign of weakness. Because I was being controlled by [opioids] and [now] I need something else to control [me]” [Participant 10]
		“I don’t see [the point of MOUDs]. You get more in the dirt. You get deep in a hole. That’s how I believe. The best thing that you can do, as a human being, is quit for yourself” [Participant 12]
		“There’s also a real element of my own pride that I’ve successfully gotten sober cold-turkey and not been on anything in the past and been able to do that for 2 years. I look at that and say, “Well, I did it then. I should be able to do it now. I shouldn’t need this crutch” [Participant 5]
		“Sometimes, some of [the 12-step sponsors] will say something to their sponsee [taking an MOUD]… Well, you’re still taking dope” [Participant 11]
	Helpful when willpower is difficult	“…would be great for [people] who don’t have the will, power, and mental acuity [to quit on their own]” [Participant 13]
		“Basically, what it came down to is I knew I didn’t have enough self-control at the time with opioids. At the time I knew Suboxone makes it better. I didn’t have to rely on only willpower and self-control, which is not something I'm particularly good at, at the time” [Participant 4]
Cues to action	Desire for OUD treatment in the mental health setting	“I think it’d be good [to get OUD treatment in the mental health clinic] because I’m getting helped in both ways, with my mental health and the use of opiates. It’s like killing two birds with one stone” [Participant 8]
		“I don’t know how much [mental health clinics] deal with the actual giving out [of] medications. I think that’d be awesome [if they did] because opioid use addiction and mental health go hand-in-hand” [Participant 10]

### HBM Constructs and Corresponding Major Themes

#### [HBM Construct: Severity/Susceptibility]: Theme: Readiness to Quit is Critical for Consideration of MOUDs

When asked about what might motivate people with OUD to consider treatment with MOUDs, participants noted the importance of being ready to quit, which was based on their perceptions of the severity of their OUD and their susceptibility to adverse outcomes such as death. Participants who successfully used MOUDs to achieve sobriety shared the experiences that led them to try the medications. These typically focused on the physical and mental stressors of taking opioids that led them to a point at which they felt compelled to stop using. As noted by one participant, they felt: “tired of being dominated by opioids, by heroin, or by pills. You’re tired. You’re mentally, physically, and emotionally tired. You want something different…and you are willing to do whatever it takes [to stop using opioids]” [Participant 10].

#### [HBM Construct: Benefits]: Theme: MOUDs are Used to Substitute for Opioids or Stave Off Withdrawal Symptoms, not for OUD Treatment

Overall, many participants understood MOUDs not as therapeutic medications but as alternative opioids. Many participants indicated that they had obtained MOUDs on the street. A few participants lacked awareness that MOUDs were used to reduce or eliminate opioid use, and thought they only were used to lessen withdrawal. Several used methadone or buprenorphine to get high or to prevent withdrawal until they were able to get more opioids. As one participant noted:

Suboxone, I used it here and there when I couldn’t find any [opioids]. That’s always the first thing that people have a lot to give away. I never saw it as a way to stop…it would just hold you over till you can find something [Participant 3].

This sentiment was prevalent among those who were familiar with MOUDs. One participant summed up their knowledge about MOUDs by calling them “a cheap way of getting high” and spoke about methadone as a way to “still go use [opioids] a little bit” [Participant 2]. Some observed that methadone clinics were a good place to obtain methadone for getting high when they did not have enough money to buy opioids. As one stated:

To tell you the truth, when I didn’t have money [the methadone clinic is] where I would go to get my fix on. I would get the methadone and that would keep me cool until I do get money to go get heroin. So it really wasn’t that I was trying to get off [heroin], it was just that I needed [methadone] to keep my day going [Participant 8].

Perhaps due to these perceptions and experiences, some participants voiced disbelief regarding the effectiveness of MOUDs for promoting sobriety. A few spoke about needing to feel withdrawal symptoms in order to gain motivation to reduce opioid use. As one stated: “I was like, ‘Oh no, this is such a crutch. No one’s going to get sober like that.’ They’ll take it one day and be sober maybe for that day, but they don’t feel the withdrawal, so they go onto using the very next day” [Participant 3]. Another participant echoed these thoughts by saying: “The pain of the withdrawals is what keeps you from wanting to do it again. If you take the experience of the pain out of it, you don’t learn the lessons” [Participant 13]. However, some voiced support for MOUDs, indicating that it was comforting to know that “I wasn’t going to go through withdrawals or the pain of withdrawal sickness” if MOUDs were taken [Participant 4] and that “If I’ve got something that can take away the sickness [of withdrawals] then I’m all for it” [Participant 5].

#### [HBM Construct: Costs as Harm or Burden]: Theme: Negative Perceptions About Methadone and Methadone Clinics are Prevalent

When asked about what they had heard about MOUDs, many participants immediately started talking about the costs of MOUDs in terms of their harms or burdens and their negative perceptions of methadone. These included complaints about the physical side effects. “Methadone, it’s not a good medicine because it messes all your calcium, your bones, all your body…you become an old man” [Participant 12]. Others spoke about its lack for effectiveness for achieving sobriety, as it did not stem their craving for opioids, and sometimes led to increased opioid use. One participant noted, “I did take methadone for about 2 years. It didn’t help…I used more heroin” [Participant 7].

A common sentiment was that methadone clinics were not patient-centered. Clinic inflexibility regarding the time of methadone administration allowed for no leeway if people were late due to unforeseeable events such as car problems. Others talked about the poor treatment received from clinic staff. As exemplified by one participant:

I feel like sometimes the [methadone clinic] staff is not knowledgeable on the way that [an addict] might feel. The patient or person seeking help might snap in terms of getting mad or very irritated or seeming like in a rush. I would see sometimes that the staff would make them even wait longer. I would just say – just toy with their addiction, and that really makes you not want to go back there [Participant 6].

One participant with a history of methadone use summarized it this way:

I personally used methadone and I do not recommend it at all. It’s 10 times more addicting than heroin. The withdrawal is worse than heroin as well. For some reason, clinics only like to give you dosage in the early, early morning, one location. If you miss that dose, you’re almost doomed to have to go use because of that withdrawal, because of that craving. It’s like the lesser of the evil would be heroin in my eyes. I don’t recommend methadone. Would never use that again [Participant 3]

These views about methadone made about half of the participants (n = 6) wary about other MOUDs, but 3 others disclosed that they had success with non-methadone MOUDs. Two participants were unaware of the existence of other MOUDs.

#### [HBM Construct: Locus of Control]: Theme: MOUD Use Feels Like “Cheating”

Participant views about their control over their opioid use (known as locus of control, which can be seen as internal, within oneself, coming from an external source, or related to chance) influenced their perceptions about MOUD. Ten of 13 participants perceived MOUD use not as quitting opioids, but rather as switching from one substance to another. These participants did not view MOUDs as a way to gain control over opioid use; instead, MOUDs continued their dependence on opioids. One participant bluntly remarked: “If you’re taking that, you’re not clean”, and noted that they did not want to “substitute dope for dope” [Participant 13].

Many participants described recovery as requiring an internal locus of control, voiced their belief that recovery from opioid use requires an internal change that allows a person to gain control, and viewed MOUD use as a form of relinquishing control. For these participants, sobriety requires self-control and willpower, not medication aimed at lessening use. Some asserted that they did not believe in substituting for willpower when quitting. One individual who had several attempts at sobriety before achieving it with MOUDs recounted, “In the past, yes, I felt…you should be able to, on your own will be able to stop yourself from taking [opioids]. You don’t need [MOUDs], is what I told myself”. [Participant 5]. This participant observed that their initial resistance to MOUD use may have stemmed from their involvement in 12-step programs:

There’s this weird resistance to it. I’m not laying blame or pointing fingers, I just think I spent a lot of years in AA [Alcoholics Anonymous] and have absorbed some of the thinking that’s in the air around drugs like Suboxone [buprenorphine and naloxone] I’m not saying - …AA as an organization doesn’t have anything to say about [MOUDs] other than basically, “If these are things that you need to guarantee that you stay alive and to protect your health then that’s fine”. But there’s this undercurrent, I feel like, in the rooms of like, “Well it’s sort of like cheating, or you’re not really sober if you’re on Suboxone” [Participant 5].

Participants commonly expressed similar views about 12-step programs, with one participant noting that they worried about disclosing their Suboxone use to other members of their Narcotics Anonymous program because: “I felt like I would be considered I was not free of opiates. I would say that I wasn’t taking [Suboxone], but in reality, I was” [Participant 6]. Another participant observed: “Twelve-step programs, their definition of sober is not being on anything at all. Like anything. Ibuprofen and stuff like that” [Participant 4].

#### [HBM Construct: Cues to Action]: Theme: Desire for OUD Treatment in the Mental Health Setting

All participants in this study declared that they wanted the convenience of having their mental health clinic assess and provide general treatment for their substance disorder. As one observed:

That would be a one-stop-shop. If it was available [at their mental health clinic] that would be perfect. I do believe that…opioids go along with mental health because everything is centered in the mind. If I can treat my mind and my body in one place, then that would be nice. That would be nice. It would cut out travel time, money, gas [Participant 1].

Others also noted that mental health and OUD “go hand in hand” [Participants 10, 13] and spoke about the importance of treating both at the same time.

Many participants lauded their relationships with their public mental health clinic therapists and substance use counselors. Many noted that they felt more open to discussing their opioid use with these clinicians than with their psychiatrists, though most did not mention discussing MOUDs with their therapists or counselors. One participant disclosed: “She’s my therapist, and there’s a trust that is developed between her and I, and because of that trust between her and I, I’m slowly peeling like the onion and exposing the secret stuff, the stuff that I would normally not talk about” [Participant 10].

All participants were enthusiastic about the prospect of receiving care at their mental health clinics due to convenience, the benefits of treating both mental health and OUD simultaneously, and the trust their placed in their therapists and counselors. However, a few expressed uncertainty about whether their mental health providers had expertise with MOUDs. One participant observed, “I don’t know [if my psychiatrist] can even prescribe me [MOUDs]. I’m not sure. Maybe she can. I’m not really sure, in terms of where her capabilities are” [Participant 3]. Several others remarked that they were unsure about the scope of their mental health providers’ practice, since OUD and MOUDs were not raised during their visits.

### Minor Themes

Several themes mentioned by fewer than half of the participants centered on concerns regarding the MOUDs themselves. These included concerns about interactions between psychiatric medications and MOUDs, as well as potential side effects. A few participants remarked that ongoing use of MOUDs was undesirable. Instead, they preferred taking a medication only for a short period to aid with withdrawal symptoms. As noted by one participant: “You’re supposed to give somebody something to wean them off, not to keep them on…now these people get [MOUDs] all the time. I totally don’t like that” [Participant 11].

Participants with experience taking non-methadone MOUDs perceived the medications positively. Participants noted benefits including not experiencing withdrawal symptoms, not needing to take so many pills due to the long-acting nature of the MOUDs, and not needing to rely solely on willpower and self-control.

### Putting It Together: Perceptions of MOUD in the Context of the Health Beliefs Model (HBM)

One participant’s experience with Suboxone illustrates relationships between the constructs of the HBM. This individual was seeing a psychiatrist and a highly trusted therapist for depression at a public mental health clinic. The participant recognized the *severity* of his condition and his *susceptibility* to adverse outcomes, as he admitted that “I’m an IV heroin user and that relapse can end in death or in other terrible health consequences”. He initially resisted receiving a prescription for MOUD because of the *costs*, noting, “I wasn’t all that into the idea of taking [it] for the long term or having it prescribed”. Yet he appreciated the *benefits* of using Suboxone to avoid withdrawal symptoms. He viewed MOUDs as facilitating self-control or reinforcing *internal locus of control*, so he obtained Suboxone illicitly, and attempted “to taper myself down really quickly and just try to get completely clean”. His perceptions of the severity/susceptibility of his problem increased when he “ended up pretty strung out again on heroin for several days”. At this point, *cues to action* by his psychiatrist, who while treating his depression, “the entire time…is suggesting that Suboxone or Naltrexone, either of these are an option”, moderated his need for maintaining internal locus of control, and resulted in his request for an MOUD prescription [Participant 5]. The relationship between these constructs elucidate potential drivers of MOUD acceptance and use among people with mental illness and OUD.

## Discussion

This study collected in-depth qualitative data from past or current OUD clients regarding their perceptions about MOUD and preferences for receiving MOUD treatment in public mental health settings. This study adds to existing studies on perceptions of MOUDs (Cioe et al., 2020; Oliva et al., 2011; Randall-Kosich et al., 2020; Silverstein et al., 2019; Yarborough et al., 2016). However, uniquely, we explored perceptions of people with mental illness receiving care in public mental health settings and examined these perceptions using the HBM framework to advance understanding of the fundamental dynamics underlying demand for MOUDs.

Consistent with our prior study on MAUD treatment (Bromley et al., 2020), we found that the HBM provides a good framework for understanding decisions about MOUD use. For both types of substances, readiness to quit is a precursor for consideration of pharmacologic treatment (Bromley et al., 2020; Pettersen et al., 2018). In contrast to patients with alcohol use disorder, patient evaluation of the benefits and costs of MOUDs may be more strongly influenced by their prior experiences. We found that since MOUDs are readily obtained on the streets, participants often perceived that they were not a means for reducing opioid use. Instead, participants saw MOUDs as another opioid to help them get high or as a method to stave off withdrawal symptoms. Since the strongest predictor of willingness to take MOUDs is believing that they are effective for stopping opioid use (Ober et al., 2021), there is a need to educate patients about the role of these medications in opioid cessation.

Once people were ready to quit, their views regarding control over their OUD mediated their views of the benefits and harms of the medication. For instance, many participants valorized a view that recovery should be achieved “on your own”, and expressed concerns that choosing to use MOUDs would undermine their ability to control their recovery. Because many participants believed quitting required or was best achieved by strengthening one’s self-control or other internal resources like willpower or motivation to quit, the perception that MOUDs increase one’s control over opioid use increased acceptance of the medications. In addition, those who viewed MOUDs as a means of increasing self-control during periods of withdrawal, such as those who disclosed that they had experienced difficulties quitting cold turkey and those who believed that MOUDs were beneficial for staving off withdrawal symptoms, tended to have greater receptiveness to MOUD use. So for some, external locus of control was acceptable. Alternatively, those who strongly believed that MOUDs constituted “cheating” (and therefore believed that they undermined individual locus of control), who believed in the need to experience withdrawals, or who lacked recognition of the benefits of MOUDs, were reluctant to take them. Several participants who remarked that they had quit opioids without any pharmacologic assistance spoke about their belief in the value of experiencing withdrawals and in the importance of self-control without pharmacologic assistance.

Our findings support having public mental health clinics provide OUD treatments, because as trusted and available providers they may serve as a stimulus or “cue to action” for MOUD use. Individuals in this study strongly supported the convenience of receiving OUD treatment and of information about MOUDs from their psychiatrists, therapists, and substance use counselors. Many had strong relationships with their therapists and substance use counselors and felt comfortable sharing potentially stigmatizing details about their substance use and efforts at sobriety.

Study participant uncertainty about whether their mental health providers had experience with OUD or MOUDs underscores the need for mental health providers to more proactively approach and inquire about opioid use in patients with mental illness and to discuss potential pharmacologic treatments with them and reinforce the effectiveness of MOUD for treating OUD. Psychiatrists can address the unique concerns patients with mental illness regarding potential MOUD-psychiatric medication interactions. Therapists and substance use counselors could be trained to engage patients with mental illness in these discussions and to refer interested individuals to psychiatrists for further information.

Most participants were familiar with methadone, but had little or no knowledge about other MOUDs. Mirroring findings from previous studies (Cioe et al., 2020), we found that participant perceptions of methadone were generally negative. These negative views may color patient perceptions about and willingness to consider other MOUDs, particularly when coupled with poor knowledge about other less well-known MOUDs (Weicker et al., 2019). Our results indicate the critical role for mental health clinicians to educate patients about the different MOUD formulations, their benefits, and effectiveness for OUD treatment.

### Strengths and Limitations

To the best of our knowledge, this study is the first to examine patient perspectives of MOUD among in individuals with a co-occurring mental health disorder. Prior studies also have not included vulnerable patients receiving care in a publicly-funded mental health system. This study has several limitations.. The study included a small number of participants. However, even with this small number, we reached saturation of themes (no new themes observed) after 7 interviews, suggesting that additional interviews may not have yielded additional information. Further, many study participants had already stopped using opioids, so their views about MOUDs were influenced by their chosen method of OUD cessation, including MOUD. Lastly, because participants were invited to participate by their clinicians, selection bias may have occurred.

## Conclusion

In conclusion, patients raised multiple obstacles that clinicians need to be aware of and address when raising the potential for MOUD treatment. These include patient perceptions that MOUDs are used not for treatment, but rather as a substitute for opioids or as a temporary solution to help stave off withdrawal symptoms, negative previous experiences with methadone and methadone clinics that influenced their perceptions of MOUDs, and views that people using MOUDs were “cheating”, and were not completely clean. Of promise though, patients with co-occurring mental health and opioid use disorders were receptive to learning about and receiving naltrexone and buprenorphine MOUD from their clinicians in public mental health clinics, though they mostly reported having minimal discussions about MOUD with their mental health clinicians. This is likely because the setting in which this study was conducted has low MOUD prescription rates. This study supports the need to expand the implementation of strategies that support uptake of MOUD (e.g., screening, counseling and education, prescriber supports) for patients with co-occurring disorders seen in mental health clinics.
